# Actuator-Driven, Purge-Free Formaldehyde Gas Sensor Based on Single-Walled Carbon Nanotubes

**DOI:** 10.3390/nano15130962

**Published:** 2025-06-21

**Authors:** Shinsuke Ishihara, Mandeep K. Chahal, Jan Labuta, Takeshi Tanaka, Hiromichi Kataura, Jonathan P. Hill, Takashi Nakanishi

**Affiliations:** 1 Research Center for Materials Nanoarchitectonics (MANA), National Institute for Materials Science (NIMS), 1-1 Namiki, Tsukuba 305-0044, Ibaraki, Japan; 2 School of Chemistry and Forensic Science, University of Kent, Canterbury CT2 7NH, UK; 3 NMR Spectroscopy Group, Institute of Organic Chemistry and Biochemistry (IOCB), Czech Academy of Sciences (CAS), Flemingovo nám. 2, 160 00 Prague, Czech Republic; 4 Research Institute of Core Technology for Materials Innovation, National Institute of Advanced Industrial Science and Technology (AIST), Tsukuba 305-8565, Ibaraki, Japan; 5 Nanomaterials Research Institute, National Institute of Advanced Industrial Science and Technology (AIST), Tsukuba 305-8565, Ibaraki, Japan

**Keywords:** gas sensor, semiconductor, chemiresistor, formaldehyde, carbon nanotube

## Abstract

Formaldehyde vapor (HCHO) is a harmful chemical substance and a potential air contaminant, with a permissible level in indoor spaces below 0.08 ppm (80 ppb). Thus, highly sensitive gas sensors for the continuous monitoring of HCHO are in demand. The electrical conductivity of semiconducting nanomaterials (e.g., single-walled carbon nanotubes (SWCNTs)) makes them sensitive to chemical substances adsorbed on their surfaces, and a variety of portable and highly sensitive chemiresistive gas sensors, including those capable of detecting HCHO, have been developed. However, when monitoring low levels of vapors (<1 ppm) found in ambient air, most chemiresistive sensors face practical issues, including false responses to interfering effects (e.g., fluctuations in room temperature and humidity), baseline drift, and the need to apply a purge gas. Here, we report an actuator-driven, purge-free chemiresistive gas sensor that is capable of reliably detecting 0.05 ppm of HCHO in the air. This sensor is composed of an HCHO→HCl converter (powdery hydroxylamine salt, HA), an HCl detector (a SWCNT-based chemiresistor), and an HCl blocker (a thin plastic plate). Upon exposure to HCHO, the HA emits HCl vapor, which diffuses onto the adjacent SWCNTs, increasing their electrical conductivity through *p*-doping. Meanwhile, inserting a plastic plate between HA and SWCNTs makes the conductivity of SWCNTs insensitive to HCHO. Thus, via periodic actuation (insertion and removal) of the plastic plate, HCHO can be detected reliably over a wide concentration range (0.05–15 ppm) with excellent selectivity over other volatile organic compounds. This actuator-driven system is beneficial because it does not require a purge gas for sensor recovery or baseline correction. Moreover, since the response to HCHO is synchronized with the actuation timing of the plate, even small (~0.8%) responses to 0.05 ppm of HCHO can be clearly separated from larger noise responses (>1%) caused by interfering effects and baseline drift. We believe that this work provides substantial insights into the practical implementation of nanomaterial-based chemiresistive gas sensors.

## 1. Introduction

Formaldehyde vapor (HCHO) is a harmful pollutant that continuously evaporates from construction materials (e.g., adhesives, resins, paints, and preservatives) in the interiors of buildings and constitutes a health hazard leading to severe conditions, including dermatitis, asthma, and cancer [1]. For this reason, the World Health Organization (WHO) has set the permissible level of HCHO indoors below 0.08 ppm (80 ppb) [2]. Although conventional analytical methods such as hydrazine-labeled liquid chromatography and detector tubes can be used for the quantification of HCHO [3,4], its continuous monitoring is made inconvenient by requirements including the frequent exchange of consumable items, individual manual sampling, or cumbersome instruments. Thus, a demand for advanced gas sensors with excellent sensitivity, selectivity, portability, cost-effectiveness, low power consumption, and continuous operability has emerged [5,6,7,8].

Of the various potential detection mechanisms, chemiresistive gas sensors that monitor the variation in electrical conductivity in semiconducting materials are ideal for integration into portable devices (e.g., smartphones and IoT devices) due to their simple configurations [9,10,11,12,13]. The electrical conductivity of semiconducting nanomaterials (e.g., sorted single-walled carbon nanotubes (SWCNTs) [14,15]) can be affected by chemical substances adsorbed onto their surfaces, and highly sensitive and selective chemiresistive gas sensors (including for HCHO sensing [16,17,18,19,20,21,22,23,24]) have been developed by using post-synthesis chemical functionalization [25,26,27,28,29,30]. However, when monitoring very low levels of trace gases (<1 ppm) in ambient air, most chemiresistive sensors face practical issues, including false responses to sensing conditions (e.g., fluctuations in room temperature and humidity), baseline drift during long-term operation [31], or the requirement for a purge gas for sensor recovery and baseline correction. These issues arise largely a consequence of a material’s weak sensing response to low concentrations of gas-phase analytes, making it difficult to establish an applicable signal-to-noise ratio.

We recently reported an HCHO sensor comprising a non-deliquescent hydroxylamine salt sandwiched between membrane filters (HA) and an SWCNT network embedded in a comb-shaped gold electrode (SWCNT-based chemiresistor) [32]. HA and SWCNTs are spatially isolated (ca. 0.4 mm), and SWCNT-based chemiresistors monitor the diffusion of HCl vapor resulting from the condensation of HCHO and HA. Since HA is present in excess compared to the ppm-level HCHO analyte, the sensor can be used to continuously and repetitively monitor HCHO with confirmed operational stability for 6 months. However, the sensor continuously reacts with HCHO, so purging air (i.e., with HCHO-free air) is required for baseline correction and recovery. Although high-pressure gas cylinders or purified air can be used for purging, this requirement limits continuous operation and negatively affects the dimensions of the sensor system as a whole.

**Figure 1:**
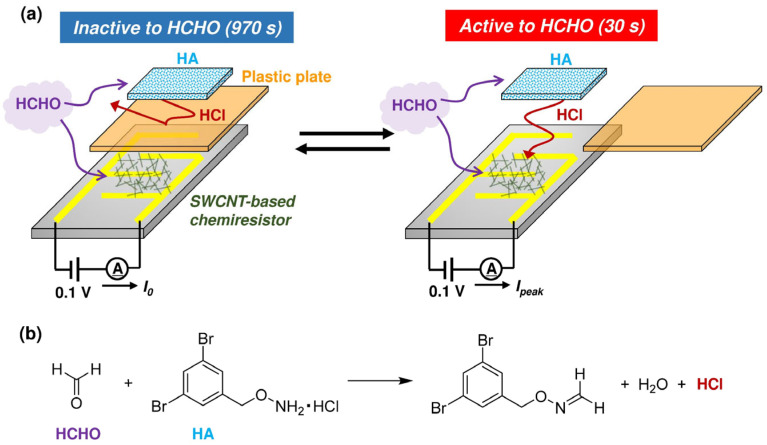
Actuator-driven, purge-free chemiresistive HCHO sensor used in this work. (**a**) HCHO sensor composed of a powdered hydroxylamine salt sandwiched between hydrophobic polytetrafluoroethylene (PTFE) membrane filters (HA), an SWCNT network embedded on a comb-shaped gold electrode (SWCNT-based chemiresistor), and a thin plastic plate. The HA and SWCNT-based chemiresistor are spatially isolated, and the intervening plastic plate is periodically removed to monitor HCHO in the air. Note that the sensor was only exposed to analyte gases without switching to purge gas. (**b**) Condensation reaction between HCHO and HA, generating HCl vapor.

In this work, we demonstrate an actuator-driven, purge-free chemiresistive HCHO sensor realized by the introduction of a thin plastic plate into our abovementioned sensor [32] (Figure 1a). When a plastic plate is inserted between HA and SWCNT, the SWCNT-based chemiresistor is insensitive to HCHO since HCl vapor is no longer able to access the chemiresistive region, thus making sensor recovery and baseline setting possible even in the presence of HCHO in the surrounding air. Moreover, since the response to HCHO is synchronized with the actuation timing of the plate’s removal, small responses (~0.8%) to 0.05 ppm of HCHO can be separated from larger noise responses (>1%) caused by interfering effects and baseline drift. Thus, through periodic actuation (insertion and removal) of the plate, HCHO can be reliably detected over a wide concentration range (0.05–15 ppm), with excellent selectivity over other volatile organic compounds (VOCs). As a general concept, an actuator-driven chemiresistive sensor consists of a selector component (in this case, HA), a detector component (in this case, a SWCNT-based chemiresistor), and an actuator component (in this case, the plastic plate). The selector and detector components are spatially isolated. The actuator enables temporary cessation of selector–detector interactions, so sensor recovery and baseline corrections are possible without the need to apply a purge gas. We believe that this work provides substantial insights into the practical implementation of nanomaterial-based chemiresistive gas sensors, particularly for detecting low-level trace gases in real-world environments.

## 2. Experimental Section

### 2.1. Materials

Chemicals and solvents were purchased from commercial sources and used as received unless otherwise stated. Purified water (18.2 MΩ cm) was obtained from a Milli-Q system (Direct-Q 3 UV, Merck (Tokyo, Japan)). Formalin solution (37 wt%) and 2-butanone were obtained from TCI Chemical Co., Ltd. (Tokyo, Japan). Acetaldehyde was obtained as a permeation tube (P-92-1) from GASTEC (Kanagawa, Japan). Methanol, ethanol, tetrahydrofuran (THF), ethyl acetate, toluene, and hexane were obtained from Fujifilm Wako Pure Chemicals Co., Ltd. (Osaka, Japan). Acetone and chloroform-d were obtained from Kanto Chemical Co., Ltd. (Tokyo, Japan).

### 2.2. Preparation of the HCHO Sensor

SWCNT network chemiresistor was prepared by using semiconducting SWCNTs (sorted using the column chromatography technique) (Figure S4) [33], a dispersing agent based on metallo-supramolecular polymer (Figure S5) [34,35], and interdigitated gold electrode with a gap of 200 μm (CC1.W1, BVT Technologies (Strážek, Czech Republic)) (see our previous report for details [32]). As the hydroxylamine salt (HA), we employed *o*-3,5-dibromobenzylhydroxylamine hydrochloride (see Figure 1b) due to its excellent correlation between sensing response and HCHO concentration as well as its confirmed stability for 6 months [32]. HA solid was ground using a mortar and pestle, and the powdered material (ca. 1 mg) was sandwiched between hydrophobic PTFE membrane filters (pore size: 3 μm, T300A047A, Advantec (Tokyo, Japan)). The HA patch was affixed to the SWCNT-based chemiresistor. A thin plastic plate (polypropylene, thickness = 0.20 mm) was inserted between the HA patch and SWCNT-based chemiresistor to block HCl vapor generated by the condensation reaction between HCHO and HA.

### 2.3. Generation of HCHO and Acetaldehyde Vapors

HCHO vapor was generated according to our previously reported methods [22,32]. Briefly, formalin solution (37%) was diluted with water; then, formalin solution (1 mL; individually for 37%, 7.4%, 1.85%, 0.46%, 0.123%, and 0% (wt%)), in a glass test tube, was placed in a two-necked flask (200 mL) maintained at 40 ± 1 °C. Relative humidity (RH) was controlled by blending dry and water-bubbled air by using a dry-air generator (P4-EFA, IAC (Kanagawa, Japan)), mass-flow controllers (CUBE MFC-1005-4S2-2L-AIR, FCON (Kochi, Japan)), and a humidity sensor (HMI41, VAISALA (Tokyo, Japan)). Humidity-adjusted air (50% RH, 0.3 L/min) was continuously injected into the flask as a carrier gas. The HCHO concentration was determined using a formaldehyde analyzer (FP-31, RIKEN KEIKI (Tokyo, Japan)). The HCHO concentrations obtained using this method were 14.6, 3.8, 1.2, 0.27, 0.05, and 0 ppm for each formalin solution. Acetaldehyde vapor was generated as follows. Permeation tubes (GASTEC, P-92-1) containing neat acetaldehyde liquid were placed in a two-necked flask (200 mL) maintained at room temperature (20 ± 2 °C). Humidity-adjusted air (50% RH, 0.3 L/min) was injected into the flask as a carrier gas. Acetaldehyde concentration was determined by using a detector tube (GASTEC, 92L/92M). A steady concentration of 2 ppm of acetaldehyde vapor was obtained using this method. The 2 ppm acetaldehyde vapor was diluted with humidity-adjusted air to control its concentration.

### 2.4. Generation of VOC Vapors

When generating VOC vapors, the same apparatus we used for HCHO vapor generation (diffusion tube method) was used, but this time, we used neat VOC liquid (1 mL) in a glass test tube located in the flask (except for acetone and 2-butanone). At constant flow rate and temperature (typically 0.3 L/min and 40 °C), the mass of analyte liquid in the test tube was measured, typically at 1, 2, and 3 h, and the average evaporation rate of the analyte was calibrated based on the decrease in mass. When the vapor pressure of the analyte was low, the measurement interval of mass was extended (e.g., 24 h). When detector tubes (methanol, ethanol, acetone, and 2-butanone) were available, the concentration of analyte vapors was measured to confirm that mass changes were consistent. In the cases of acetone (10, 50 ppm) and 2-butanone (8.4, 42 ppm), neat liquids were diluted with water 150 or 30 times. VOC vapors in air (50% RH) were continuously injected into the sensor chamber.

### 2.5. Evaluation of Sensors

The SWCNT-based chemiresistor was connected to a potentiostat (EmStat, PalmSens (Houten, The Netherlands)), and the electric current under constant voltage (0.10 V) was recorded using PSTrace software (v. 4.8). This low operation voltage (0.10 V) is energetically economical. Analyte gas (20 ± 2 °C, 50% RH, 0.3 L/min) was continuously injected into the sensor chamber. In sensing experiments, the thin plastic plate between the HA and SWCNT-based chemiresistor was periodically actuated by using a programmable syringe pump (YSP-201, YMC Co., Ltd. (Kyoto, Japan)) (see Figure 1a and Figure S1 for details). Typically, the plastic plate was removed for 30 s every 1000 s.

## 3. Results and Discussion

**Figure 2:**
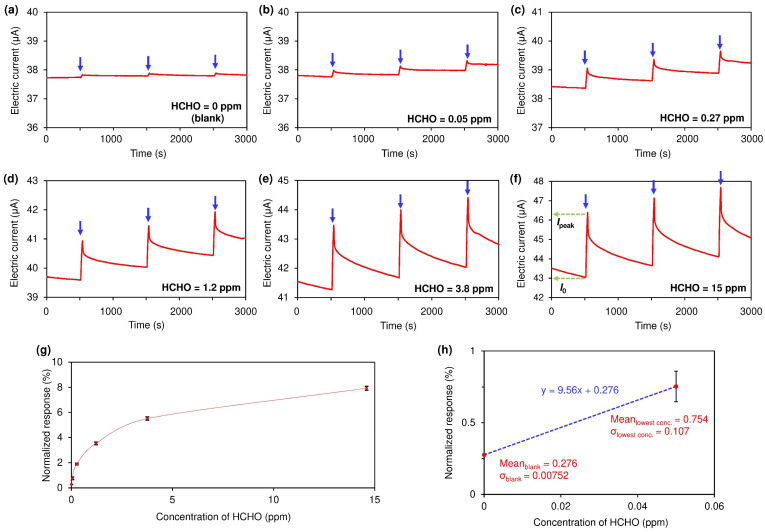
Sensing responses for HCHO at different concentrations: (**a**) blank air (50% RH), (**b**) 0.05 ppm, (**c**) 0.27 ppm, (**d**) 1.2 ppm, (**e**) 3.8 ppm, and (**f**) 15 ppm. Temporary removal (30 s) of the thin plastic plate was repeated every 1000 s (three times per run as indicated by the blue arrows). (**g**) Calibration curve: HCHO concentration versus normalized sensing response (mean of three repeated measurements with standard deviations). See Figure S2 and the corresponding test in SI for details on the calibration curve’s construction. (**h**) Normalized responses for blank air and 0.05 ppm of HCHO used for LoD estimation.

Under continuous exposure to HCHO in air (50% RH), the thin plastic plate located between the HA and SWCNT-based chemiresistor was periodically removed for 30 s every 1000 s (Figure 1a and Figure S1). As the HCHO concentration increases, the electric current at the SWCNT-based chemiresistor also increases due to *p*-doping with HCl (Figure 2a–f) [36,37,38]. Insertion of the plastic plate leads to the recovery of the original electric current even in the presence of HCHO because of the desorption of HCl from the SWCNT surface. Thus, regeneration of the sensor is possible without using a purge (i.e., HCHO-free) gas. The normalized response (%) can be calculated from Equation (1).

Normalized response (%) = (*I*_peak_ − *I*_0_)/*I*_0_ × 100, (1)

where *I*_0_ denotes the electric current before the removal of the plate, and *I*_peak_ denotes the peak electric current after the removal of the plate (see Figure 2f for details). Generally, *I*_0_ refers to the baseline electric current obtained under purge air conditions (i.e., in the absence of the target analyte). However, our actuator-driven system offers *I*_0_ values even in the presence of HCHO in the surrounding air. Thus, the normalized response and the concentration of HCHO correlate well, allowing a calibration curve for the quantification of HCHO concentration from the sensing response to be constructed (Figure 2g and Figure S2).

The limit of detection (LoD) of our sensor is estimated to be 0.020 ppm based on the calibration curve in the minimum concentration range (Figure 2h) and on Equation (2) [39].

LoD = mean_blank_ + 1.645 × σ_blank_ + 1.645 × σ_lowest conc._, (2)

where mean_blank_, σ_blank_, and σ_lowest conc._ represent the mean response for the blank, the standard deviation of the response for the blank, and the lowest gas concentration (in this case, HCHO = 0.05 ppm), respectively. The LoD of the response is estimated to be 0.46%, which corresponds to 0.020 ppm of HCHO based on the initial response (*y* = 9.56 *x* − 0.276).

We also tested a longer removal time (180 s) of the plastic plate for 0.05 ppm of HCHO, although the signal ratio of 0.05 ppm of HCHO and blank air did not improve (Figure S3). Longer removal of the plate probably leads to accumulation of the sensing response (cumulative adsorption of HCl on the SWCNT surface) and incomplete recovery (desorption of HCl from the SWCNT surface) within a reasonable period of time. Our previous study showed that it takes ca. 12 h for complete recovery following high-level HCHO exposure [22]. In addition, the risk of environmental fluctuations (e.g., variation in temperature and RH) during the absence of the plate is increased. Therefore, a short removal time is preferred for our actuator-driven system (as far as the sensing response for HCHO is concerned).

There is a subtle response to blank air (0 ppm), with the possible origins of this being (i) contamination of trace HCHO in the blank air (i.e., humidity-adjusted ambient air) or (ii) proton exchange equilibrium between HA and acidic gases in air (NO_2_, SO_2_, CO_2_, etc.) leading to evaporation of trace HCl vapor. Further investigations are necessary to elucidate the blank response mechanism.

**Figure 3:**
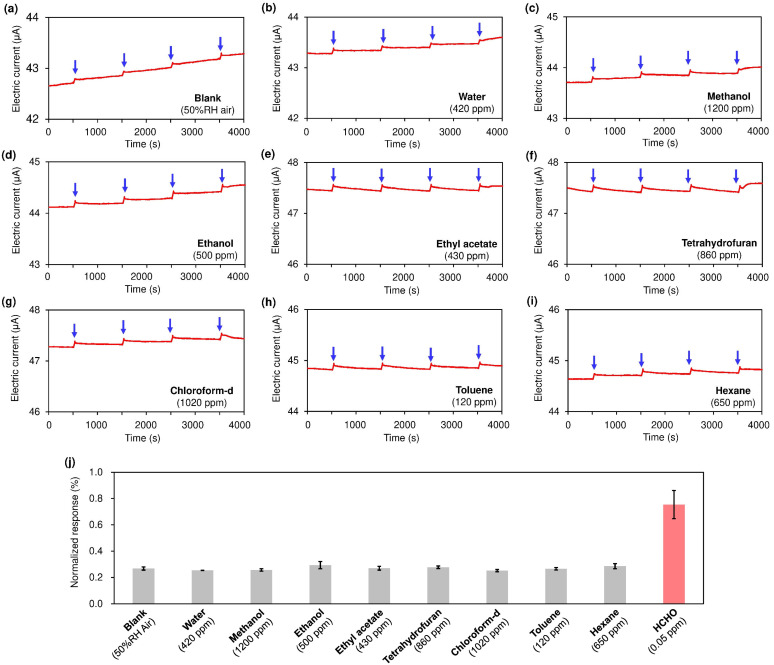
Sensing responses for non-carbonyl VOC vapors: (**a**) blank air (50% RH), (**b**) water, (**c**) methanol, (**d**) ethanol, (**e**) ethyl acetate, (**f**) tetrahydrofuran, (**g**) chloroform-d, (**h**) toluene, and (**i**) hexane. Temporary removal (30 s) of the thin plastic plate was repeated every 1000 s (four times per run as indicated by the blue arrows). Values (in ppm) quoted at the bottom right in each panel indicate the concentration of water or each VOC added into the airflow (50% RH). (**j**) Normalized sensing responses for each vapor. The average of four repeated measurements, along with their standard deviation, is shown. Since conventional chloroform (CHCl_3_) contains ethanol as a stabilizer, chloroform-d (CDCl_3_, NMR grade) was used in this study.

Figure 3 indicates that our sensor exhibits excellent selectivity for HCHO, with negligible responses to non-carbonyl VOC vapors (methanol, ethanol, ethyl acetate, tetrahydrofuran, chloroform, toluene, and hexane at 100–1000 ppm). These compounds are unreactive with HA, so the SWCNT resistor was not doped with HCl vapor.

**Figure 4:**
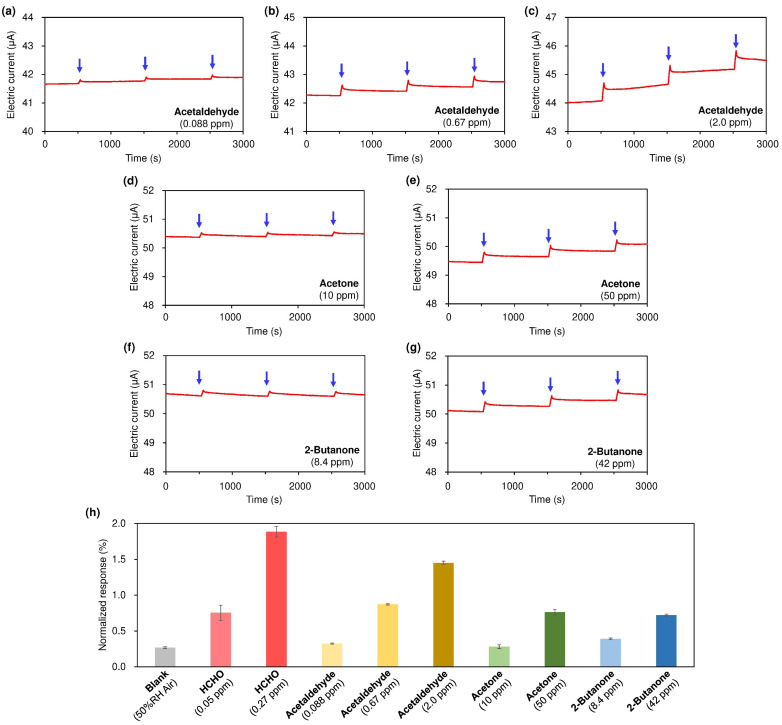
Sensing responses for carbonyl-containing VOC vapors. (**a**–**c**) acetaldehyde, (**d**,**e**) acetone, and (**f**,**g**) 2-butanone. (**h**) Normalized sensing responses for carbonyl vapors. The average of three repeated measurements, along with their standard deviation, is shown.

In contrast, carbonyl-containing VOC vapors such as acetaldehyde, acetone, and 2-butanone are reactive with HA, and our sensor exhibits responses when concentrations of these vapors are high (Figure 4a–g). However, the responses to 0.088 ppm acetaldehyde and 10 ppm acetone are close to those for blank air and appreciably smaller than those for 0.05 ppm of HCHO. The responses to 0.05 ppm of HCHO and 0.67 ppm acetaldehyde are similar, so the selectivity to HCHO over acetaldehyde is approximately 10-fold greater. Moreover, the responses to 0.05 ppm of HCHO and 42–50 ppm ketones (acetone and 2-butanone) are similar, so the selectivity for HCHO over ketones is ca. 10^3^-fold greater. The order of selectivity (HCHO > acetaldehyde >> ketones) is in good agreement with the electrophilic reactivity of aldehydes and ketones, which is weakened by the presence of *α*-alkyl groups due to steric and electron-donating effects at the carbonyl group [40].

**Figure 5:**
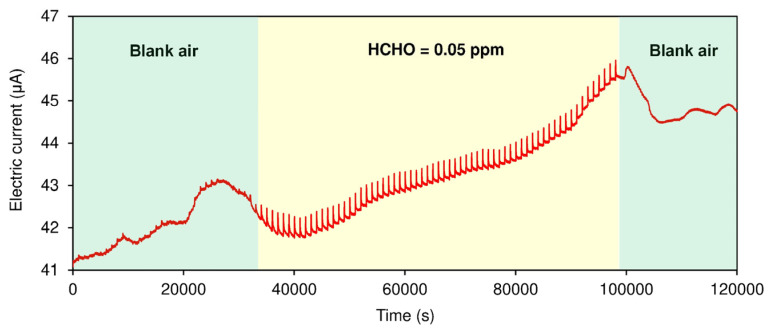
Continuous monitoring of HCHO in the air. Temporal removal (30 s) of the thin plastic plate was repeated every 1000 s in the presence and absence of HCHO.

In our actuator-driven sensor, a sensing response to HCHO should occur only when the plastic plate is intentionally removed at a known time, and the actual response to HCHO can be easily distinguished from interfering effects and baseline drift. Figure 5 shows the long-term monitoring of HCHO in air. In blank air (green region; 50% RH air), baseline drift was observed, presumably due to subtle changes in ambient temperature, but sensing responses upon periodic removal of the plastic plate (30 s removal every 1000 s) were negligibly low, indicating the absence of HCHO. When trace amounts of HCHO (0.05 ppm) were present, the actuator-driven sensor exhibited periodic spike signals synchronizing with the timing of plate removal. Even after 60 repeats, our sensor demonstrated stable performance because the amount of solid HA was much greater relative to HCHO. As soon as HCHO was cleared, the spike signals disappeared. Thus, even small responses (~0.8%) to 0.05 ppm of HCHO can be clearly isolated from larger noise responses (>1%) caused by interfering effects and unpredictable baseline drift.

It should be noted that our sensing mechanism is based on the condensation reaction between HCHO and HA and the vaporization of HCl (Figure 1), whose reaction kinetics and equilibria should be affected by the temperature and RH of the environment [22]. Thus, in practical situations, these parameters should be separately monitored for proper quantification of HCHO.

## 4. Conclusions

In conclusion, our actuator-driven, purge-free chemiresistive sensor enables the continuous monitoring of sub-ppm level HCHO, with a clear separation of noise signals due to interfering effects and baseline drift. The sensing response to HCHO is based on the nucleophilic condensation reactivity of HA to carbonyl compounds and shows excellent selectivity to HCHO over other VOC vapors. With periodic actuation (pushing/pulling motion) of the plate, sensor recovery and baseline correction are possible without requiring purging with clean air. This feature is advantageous for the miniaturization of our sensory system. This HCHO sensor is unique in that a selector component (in this case, HA) and a detector component (in this case, an SWCNT-based chemiresistor) are spatially isolated, which enables temporary cessation of selector–detector interactions via the physical insertion of the plate between the two components. Most chemiresistive sensors comprise an inseparable mixture of selector and detector components, allowing the sensor to be persistently sensitive to the target analyte. A possible solution for these sensors might involve the introduction of a filter or adsorbent (selective to the target analyte) to render the sensor temporarily insensitive to the target, leading to the construction of a different type of actuator-driven, purge-free chemiresistive gas sensor. Thus, the concept of this work can be applied generally to address some of the fundamental issues in the practical implementation of nanomaterial-based chemiresistive gas sensors. We believe this work provides a practical demonstration of the implementation of nanomaterial-based chemiresistive gas sensors, particularly useful for targeting low levels of analytes present in real environments.

## Data Availability

The data presented in this study are available upon reasonable request from the corresponding author.

## Supplementary Materials

The following supporting information can be downloaded at https://www.mdpi.com/article/10.3390/nano15130962/s1. Figure S1: (a) Photograph of HA patch (left) and SWCNT-based chemiresistive sensor (right). (b) Experimental set-up used for periodic actuation of the thin plastic plate. (c) Actuation of the thin plastic plate. Left: The thin plastic plate is inserted between HA and SWCNT-based chemiresistor, making the system insensitive to HCHO. Right: The thin plastic plate is removed from between the HA and SWCNT-based chemiresistor, making the system sensitive to HCHO. Typically, the plate insertion time is 970 s, and the plate removal time is 30 s. (d) Actuation of the thin plastic plate when HA patch is absent (provided for clarity). Left: SWCNT is covered by the plate. Right: SWCNT is not covered by the plate.; Figure S2: Fitted *y* = F(*x*) and inverse *x* = G(*y*) calibration curves. (a) HCHO concentration (*x*) to sensor response (*y*) calibration curve *y* = F(*x*). (b) Sensor response (*y*) to HCHO concentration (*x*) calibration curve *x* = G(*y*). Note that the concentration range (in ppm) for the F(*x*) calibration curve is from 0 to 15 ppm of HCHO, and the response range (in %) for the G(*y*) calibration curve is from 0 to 8%.; Figure S3: (a) Sensing responses for blank and 0.05 ppm HCHO in air (50% RH). Temporary removal (180 s) of the thin plastic plate was repeated every 1000 s (three times per run as indicated by the blue arrows). (c) Normalized sensing responses for blank and 0.05 ppm HCHO upon 30 s and 180 s removal of the plate. The average of three repeated measurements, along with its standard deviation, is shown.; Figure S4: Characterization of the SWCNT used. (a) Photograph of interdigitated electrode comprised of ceramic substrate and gold lines with a gap distance of 200 μm. (b) Photograph of the interdigitated electrode with and without SWCNT. (c–f) SEM image of interdigitated electrode embedded with SWCNT. (g) Scanning probe microscopy images of SWCNT spin-coated (3000 rpm) on flat SiO_2_/Si substrate. (h) I-V curve (0 V → 3 V → −3V → 0 V) for interdigitated electrode embedded with SWCNT (scan rate = 0.1 V s^−1^) measured under ambient air. (i) Raman spectra of SWCNTs (metallic and semiconducting) separated by column chromatography. RBM and BWF denote radial breathing mode and Breit-Wigner-Fano band, respectively. Reprinted with permission from reference [28]. Copyright 2020 American Chemical Society; Figure S5: The metallo-supramolecular polymer used as a dispersing agent of SWCNT.
