# Supplementary material for: Actuator-Driven, Purge-Free Formaldehyde Gas Sensor Based on Single-Walled Carbon Nanotubes
Source: Nanomaterials (Basel). 2025 Jun 21;15(13):962. doi: 10.3390/nano15130962 (PMC12250679; doi:10.3390/nano15130962)
Supplement: Supplementary file 1 [file nanomaterials-15-00962-s001.zip › nanomaterials-3673268-supplementary.pdf]

Supporting Information

Actuator-driven, purge-free formaldehyde gas sensor based on single-walled carbon nanotubes

Shinsuke Ishihara,[a,*] Mandeep K. Chahal,[a,b] Jan Labuta,[a,c] Takeshi Tanaka,[d] Hiromichi Kataura,[e] Jonathan P. Hill,[a] Takashi Nakanishi[a]

[a]Research Center for Materials Nanoarchitectonics (MANA), National Institute for Materials Science (NIMS), 1-1 Namiki, Tsukuba, Ibaraki 305-0044, Japan
[b]School of Chemistry and Forensic Science, University of Kent, Canterbury, CT2 7NH, UK
[c]NMR Spectroscopy Group, Institute of Organic Chemistry and Biochemistry (IOCB), Czech Academy of Sciences (CAS), Flemingovo nám. 2, 160 00 Prague, Czech Republic
[d]Research Institute of Core Technology for Materials Innovation, National Institute of Advanced Industrial Science and Technology (AIST), Tsukuba, Ibaraki 305-8565, Japan
[e]Nanomaterials Research Institute, AIST, Tsukuba, Ibaraki 305-8565, Japan

*Correspondence: ISHIHARA.Shinsuke@nims.go.jp

1. Actuator-driven sensing experiments

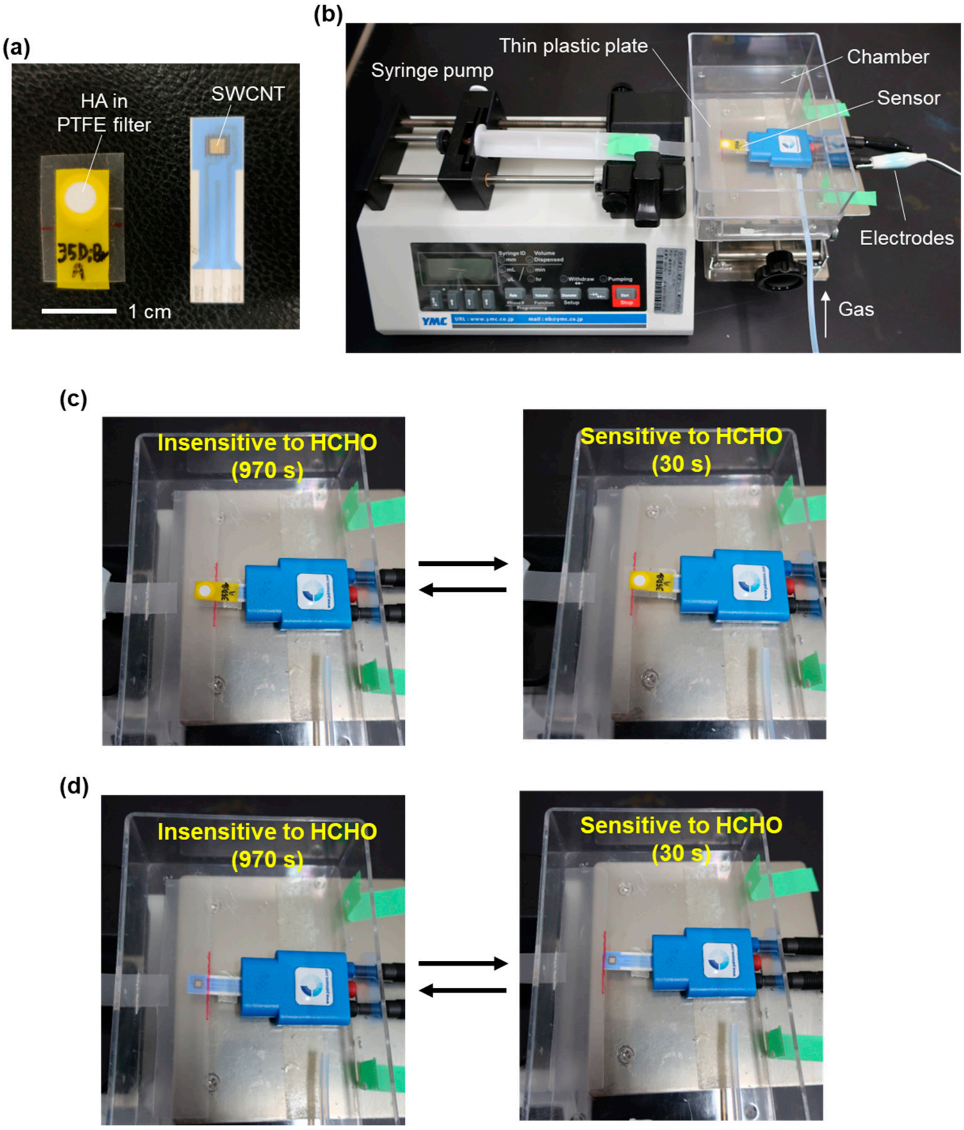

Figure S1. (a) Photograph of HA patch (left) and SWCNT-based chemiresistive sensor (right). (b) Experimental set-up used for periodic actuation of the thin plastic plate. (c) Actuation of the thin plastic plate. Left: The thin plastic plate is inserted between HA and SWCNT-based chemiresistor, making the system insensitive to HCHO. Right: The thin plastic plate is removed from between the HA and SWCNT-based chemiresistor, making the system sensitive to HCHO. Typically, the plate insertion time is 970 s, and the plate removal time is 30 s. (d) Actuation of the thin plastic plate when HA patch is absent (provided for clarity). Left: SWCNT is covered by the plate. Right: SWCNT is not covered by the plate.

2. Determination of fitted calibration curve for Figure 2g

We have selected a hyperbolic-type function with four (fitted) parameters to construct the calibration curve. The data were then fitted using the following calibration curve $y = F(x)$, which provides a relationship between HCHO concentration (in ppm; denoted as x) and the sensor response (normalized change in electric current in %; denoted as y); see Figure S2a. The actual form of the calibration curve $y = F(x)$ used is

$$y = F(x) = \frac{a}{x^b + c} + d, \qquad \text{(S1)}$$

where the values of fitted parameters are $a = -27.118$, $b = 0.658$, $c = 2.491$, and $d = 11.136$ (with $R^2 = 0.9992$).

Analogically, the inverse calibration curve $x = G(y)$, which converts the sensor response (y) to HCHO concentration (x), has the following form; see Figure S2b:

$$x = G(y) = \left(\frac{a}{y-d} - c\right)^{\frac{1}{b}}, \qquad \text{(S2)}$$

where the values of a, b, c, and d parameters are identical to those in Eq. S1 (the R^2 = 0.9986 for $G(y)$). Note that the $G(y)$ is an inversion function to $F(x)$.

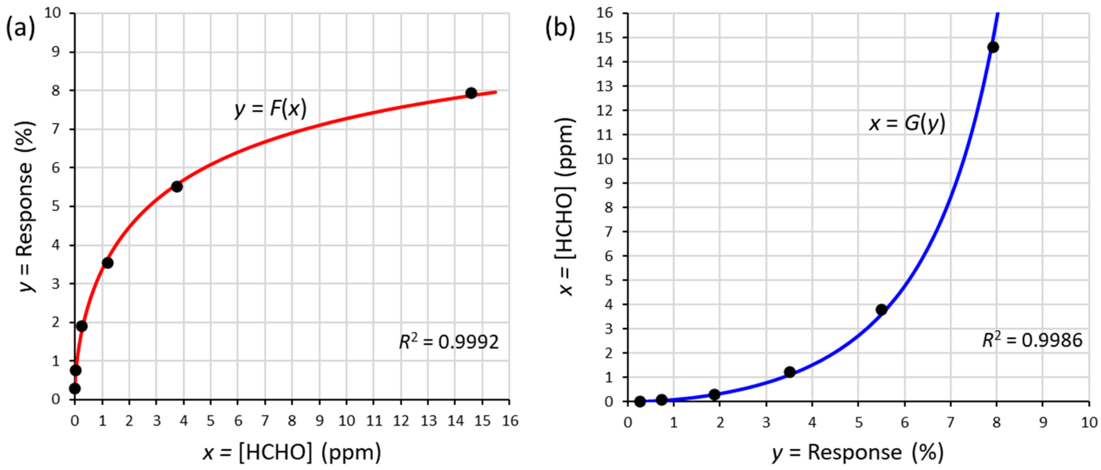

Figure S2. Fitted $y = F(x)$ and inverse $x = G(y)$ calibration curves. (a) HCHO concentration (x) to sensor response (y) calibration curve $y = F(x)$. (b) Sensor response (y) to HCHO concentration (x) calibration curve $x = G(y)$. Note that the concentration range (in ppm) for the $F(x)$ calibration curve is from 0 to 15 ppm of HCHO, and the response range (in %) for the $G(y)$ calibration curve is from 0 to 8%.

3. Longer duration removal of the thin plastic plate

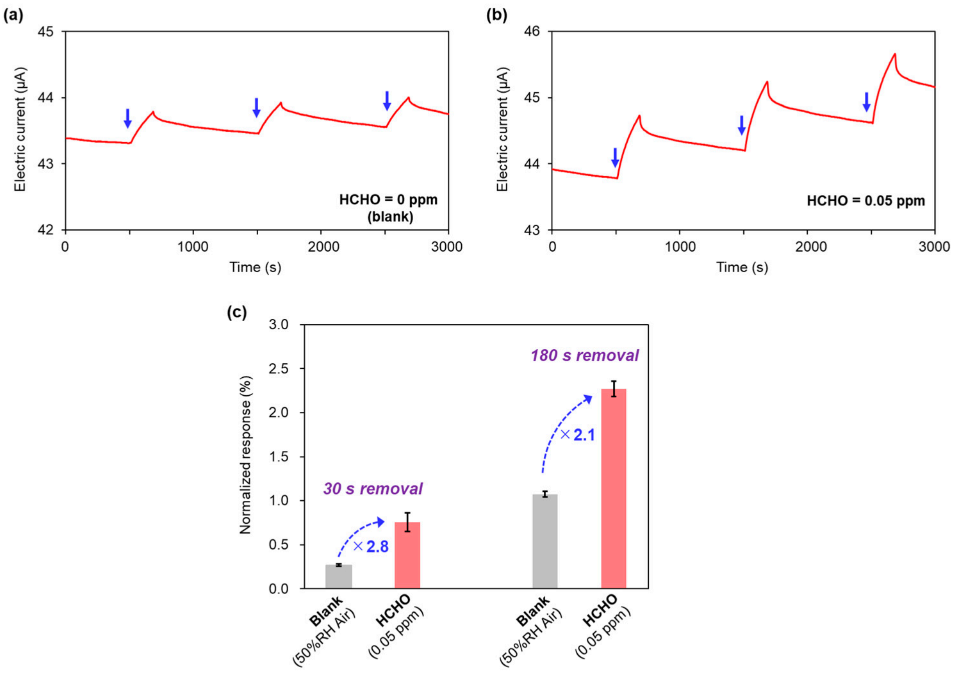

Figure S3. (a) Sensing responses for blank and 0.05 ppm HCHO in air (50% RH). Temporary removal (180 s) of the thin plastic plate was repeated every 1000 s (three times per run as indicated by the blue arrows). (c) Normalized sensing responses for blank and 0.05 ppm HCHO upon 30 s and 180 s removal of the plate. The average of three repeated measurements, along with its standard deviation, is shown.

4. Information regarding the materials used

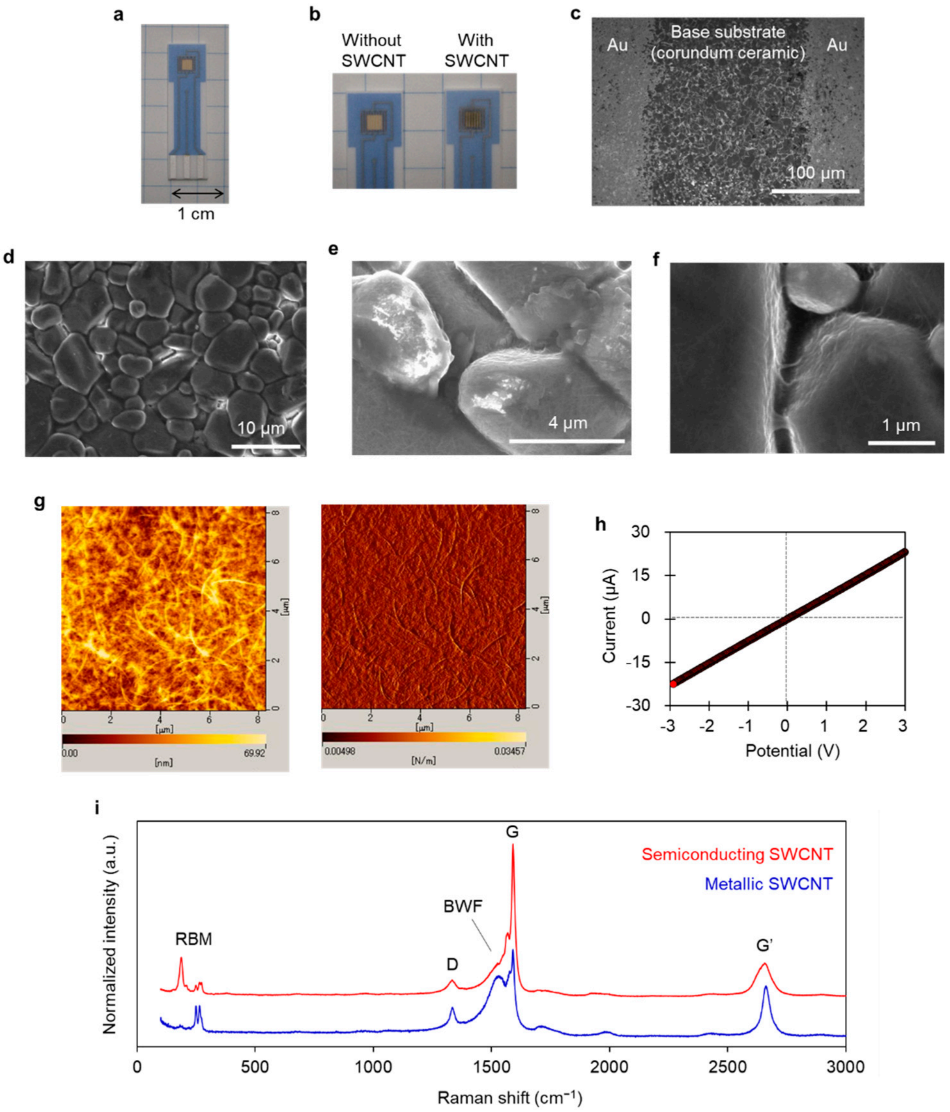

Figure S4. Characterization of the SWCNT used. (a) Photograph of interdigitated electrode comprised of ceramic substrate and gold lines with a gap distance of 200 μm. (b) Photograph of the interdigitated electrode with and without SWCNT. (c−f) SEM image of interdigitated electrode embedded with SWCNT. (g) Scanning probe microscopy images of SWCNT spin-coated (3000 rpm) on flat SiO_2/Si substrate. (h) I-V curve (0 V → 3 V → −3V → 0 V) for interdigitated electrode embedded with SWCNT (scan rate = 0.1 V s^{-1}) measured under ambient air. (i) Raman spectra of SWCNTs (metallic and semiconducting) separated by column chromatography. RBM and BWF denote radial breathing mode and Breit-Wigner-Fano band, respectively. Reprinted with permission from reference [28]. Copyright 2020 American Chemical Society.

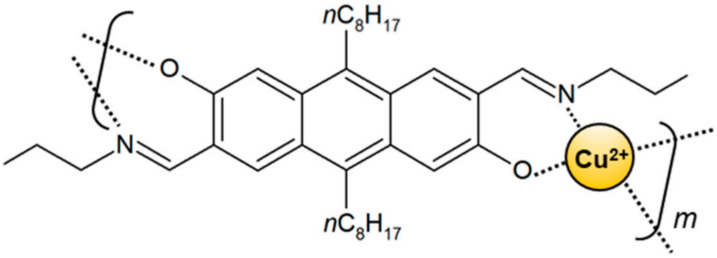

Figure S5. The metallo-supramolecular polymer used as a dispersing agent of SWCNT.
